# Psychiatric Manifestations of *ATP13A2* Mutations

**DOI:** 10.1002/mdc3.13034

**Published:** 2020-09-04

**Authors:** Bettina Balint, Joana Damasio, Francesca Magrinelli, Rita Guerreiro, Jose Bras, Kailash P. Bhatia

**Affiliations:** ^1^ Department of Clinical and Movement Neurosciences Queen Square London United Kingdom; ^2^ Department of Neurology University Hospital Heidelberg Germany; ^3^ Department of Neurology Oporto University Hospital Center Porto Portugal; ^4^ Institute for Molecular and Cell Biology, Institute for Research and Innovation in Health, Oporto University Porto Portugal; ^5^ Department of Neurosciences Biomedicine and Movement Sciences, University of Verona Verona Italy; ^6^ Center for Neurodegenerative Science, Van Andel Institute Grand Rapids Michigan USA

**Keywords:** ATP13A2, behavioural, psychiatric, vertical gaze palsy, ataxia, spasticity

## Abstract

**Background:**

Biallelic mutations in *ATP13A2* were identified as the cause of Kufor‐Rakeb disease, a pallido‐pyramidal syndrome characterized by young‐onset dystonia–parkinsonism with vertical supranuclear gaze palsy, spasticity, and cognitive decline. The phenotypic spectrum has broadened since, but predominantly psychiatric or behavioral manifestations have not been highlighted.

**Cases:**

Here we report the clinical, radiological, and genetic findings in 2 unrelated patients with *ATP13A2* mutations. One patient had a prominent behavioral (autistic spectrum) presentation and the other a psychiatric (paranoid psychosis) presentation. Both had additional features, such as delayed milestones, ataxia, pyramidal signs, upgaze restriction, or impaired cognition to varying extent, but these were partly subtle or developed later in the disease course.

**Conclusion:**

Prominent behavioral or psychiatric features can be the first or most prominent manifestation of *ATP13A2*‐related disease. They may be a diagnostic clue in patients with ataxia, spasticity, or parkinsonism and may require an interdisciplinary neurological and psychiatric treatment approach.

View Supplementary Video 1

View Supplementary Video 2

## Introduction

In 2006, biallelic mutations in *ATP13A2* were identified as the cause of Kufor‐Rakeb disease, a pallido‐pyramidal syndrome characterized by young‐onset dystonia‐parkinsonism with vertical supranuclear gaze palsy, spasticity and cognitive decline.^1^ The phenotypic spectrum has broadened since to include also hereditary spastic paraplegia (assigned locus symbol SPG 78),^2^, ^3^ neurodegeneration with brain iron accumulation,^4^ and neuronal ceroid lipofuscinosis.^5^ With the advances in neurogenetics and recent publications, it has become clear that *ATP13A2* mutations can cause a multisystem disorder affecting extrapyramidal, pyramidal, cerebellar, cortical, visual, and/or peripheral systems. Nonetheless, predominantly psychiatric or behavioral manifestations have so far not been highlighted. This can lead to diagnostic difficulty. Here we report 2 patients presenting with prominent behavioral or psychotic problems and discuss the psychiatric manifestations associated with *ATP13A2*‐related disease.

## Case Series

### Case 1

This 18‐year‐old boy was the middle of 5 children of a consanguineous couple of Pakistani origin. His antenatal and perinatal histories were unremarkable, but he had delayed motor and cognitive milestones (walking at 3 years of age, talking at 5 years of age). From the age of 11, he showed progressive gait and balance difficulties and was using a wheelchair at age 13. Cognitively, he was known to have learning difficulties, possibly also with a deterioration in adolescence, but this was not formally tested. In day‐to‐day life, one of the biggest problems was his difficulty in social interaction. Apart from the neurological symptoms, he was investigated and diagnosed in infancy with laryngomalacia, gastro‐esophageal reflux, and hypospadia. Symptomatic treatment consisted of trihexyphenidyl, baclofen, and levodopa. He had been intensely investigated (including normal brain and spine magnetic resonance imaging) without diagnosis when he presented in our clinic. The predominant feature were stereotypies, which included body rocking and pointing with the fingers, and abnormal behavior in that he did not meet the eye and tended to be reclusive (see Video S1). Although he did not speak in whole sentences, he was responsive and followed simple commands. He had hyperreflexia and a spastic gait with toe‐walking. There was dystonic posturing of the hands. He was a bit slow on finger‐tapping, but did not display clear bradykinesia. In addition, there was a mild vertical gaze palsy. A syndromic diagnosis of pallidopyramidal syndrome with cognitive difficulties and vertical gaze palsy prompted genetic testing for *ATP13A2* mutations. The patient was found to have the homozygous NM_022089.4: c.2218C > T (NM_022089.4:p.Arg740Ter) mutation of the *ATP13A2* gene. A dopamine transporter scan (DaTscan) showed bilaterally reduced availability of the presynaptic dopamine transporters. He was started on levodopa with good response but also the development of dyskinesia. Formal neuropsychometry showed prominent behavioral frontal signs and impaired executive functioning, memory performance, and praxis.

### Case 2

Case 2 is a 38‐year‐old female of Portuguese ancestry born to nonconsanguineous parents. Developmental milestones were in the normal range, but she was always clumsier and slower than other children. School performance was average, with a college degree obtained by the age of 24. From that time on, she developed a paranoid ideation, for example, that her husband was cheating on her and work colleagues were going through her notes and talked behind her back. She was later diagnosed with bulimia nervosa and had to be hospitalized as a result of severe hypoalbuminemia. At age 27, she developed psychosis with complex persecutory delusions with episodes of visual hallucinations. Antidepressants and neuroleptics were started, but even at low doses there was marked sleepiness, slowness, and clumsiness. The subsequent course was characterized by waxing and waning of the psychiatric symptoms while medication was adjusted. Eventually, treatment with aripiprazole stabilized her, but she was unable to return to work and continued to have a clumsy gait and difficulties with fine motor skills. At age 32, she had an uneventful pregnancy and a healthy baby, and came to our attention for the first time. At that time, she was taking aripiprazole 2.5 mg once daily. Examination (see Video S2) revealed facial hypomimia with frontalis overactivity. Eye movements were noticeable for upgaze restriction and a tendency to blink and use a head thrust when initiating saccades. She had a scanning dysarthria. The palmomental reflex could be elicited on the left. There were global slowness and mild bradykinesia left more than right and a mild irregular postural tremor. Pyramidal signs involved hyperreflexia with brisk jaw jerk and deep tendon reflexes, unsustained ankle clonus, and moderate leg spasticity. She had a wide‐based spastic gait without arm swing and was unable to do tandem gait.

Formal neuropsychological assessment revealed multidomain cognitive impairment with marked deficits in attention, moderate deficits in executive function and verbal memory, and mild deficits in visual memory.

**FIG. 1:**
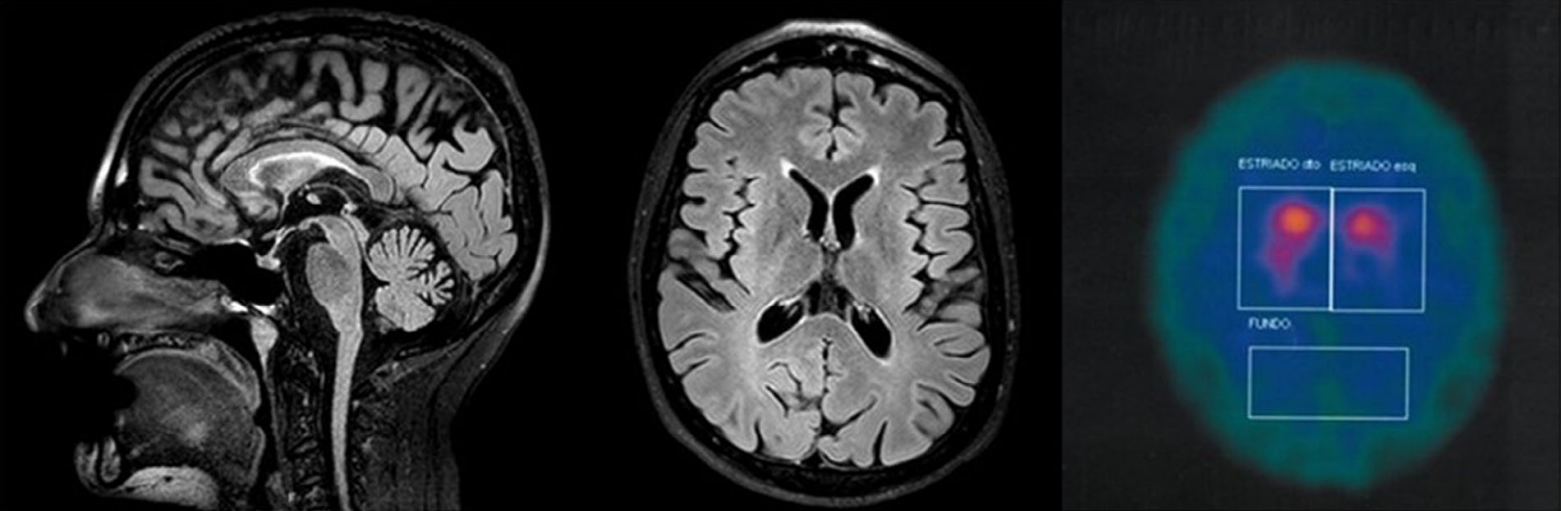
Magnetic resonance imaging and dopamine transporter scan of case 2. Sagittal (**A**) and transversal (**B**) fluid attenuated inversion recovery magnetic resonance imaging sequences show generalized brain atrophy with prominent parietal and cerebellar involvement as well as an “ear of the lynx sign” on the left (**B**). Dopamine transporter single‐photon emission computed tomography demonstrating reduced tracer uptake predominantly on the left.

Brain magnetic resonance imaging showed atrophy affecting mainly the cerebellar vermis and hemispheres and to a lesser extent both parietal lobes and the corpus callosum. Mild hyperintensity of the forceps minor of the corpus callosum was present, with a subtle “ear of the lynx” sign on the left (see Fig. 1).

A DaTscan revealed an asymmetric reduction (worse on left) of dopamine uptake (see Fig. 1). A next‐generation sequencing panel for recessive ataxias in the patient and subsequent testing of the unaffected parents disclosed 2 pathogenic variants in the *ATP13A2* gene in compound heterozygosity NM_022089.4:c.[1472_1473del];[c.2567_2568del], giving rise to 2 copies of a truncated protein (NM_022089.4:p.Gln491ArgfsTer29 and NM_022089.4:p.Pro856ArgfsTer26). Aripiprazole was stopped, resulting in only a mild improvement with regard to slowness.

After a second pregnancy, there was some deterioration in her gait, but she remained ambulatory. Subsequently, there were slowly progressive cognitive impairment and cerebellar signs evolved to include upper and lower limb dysmetria. She also developed distal lower limbs weakness. When she developed a left‐hand resting tremor and mild bradykinesia, treatment with levodopa/carbidopa 300 mg/day was started and resulted in clinical improvement. From the age of 32 until present she has remained off antipsychotics without paranoid outbreaks.

## Discussion

Here we present 2 unrelated patients with biallelic *ATP13A2* mutations who presented with prominent psychiatric and behavioral features. Case 1 manifested with prominent stereotypies and autistic‐like behavior in the context of a pallidopyramidal syndrome with cognitive difficulties and vertical gaze palsy. Case 2 came to medical attention because of psychiatric illness with paranoid psychosis and bulimia nervosa and was seen by neurologists only 8 years later with a syndrome dominated by cerebellar ataxia and spasticity.

Although theoretically these behavioral or psychiatric disturbances could have been coincidental, they are in line with previous observations of concomitant psychiatric features. For example, hallucinations, often triggered or exacerbated by medication, have been mentioned in previous reports. Visual and auditory hallucinations have been reported in the original Chilean kindred, which led to the gene discovery underlying Kufor‐Rakeb disease^6^: 2 patients had (paranoid) auditory hallucinations under treatment with trihexylphenidate, and 1 patient had visual hallucinations without medication. Recent reports of *ATP13A2* mutations manifesting as hereditary spastic paraplegia mentioned acoustic hallucinations, delusions, or paranoid ideation (without detailing their relation to medication).^3^, ^7^ Visual hallucinations and psychosis occurring during treatment with levodopa were also noted in 2 other reports.^8^, ^9^

Thus, it seems that psychosis and hallucinations are part of the clinical spectrum of *ATP13A2*‐related disease. Although in the aforementioned cases these psychiatric manifestations developed later in the disease course, in our case 2 they were the most prominent presenting feature before the neurological syndrome developed.

This observation has 2 implications. First, psychiatrists should be aware of this as another neurological cause of psychiatric illness. Second, prominent psychosis and hallucinations might be red flags and a clinical clue in the differential diagnosis of early‐onset parkinsonism, hereditary spastic paraplegia, or ataxia.

Similarly, behavioral problems have been noticed in previous reports, encompassing mainly aggression,^3^, ^8^ fatigue, and problems with drive and motivation.^3^, ^6^ Autistic spectrum presentations with stereotypies have not been described so far with *ATP13A2* mutations to the best of our knowledge, but this phenotype has been recently recognized as part of a number of early‐onset neurodegenerative syndromes, such as those seen with *WDR45* or *PLA2G6* mutations.^10^, ^11^ The latter, just as *ATP13A2*‐related and *PANK2*‐related disease, form part of the syndromes with neuronal brain iron accumulation that may manifest with prominent psychiatric features. Of note, however, the absence of iron deposition is not an exclusion of these differential diagnoses, as our cases illustrate. Lastly, Niemann Pick C is a treatable and thus not‐to‐miss differential diagnosis of early‐onset, progressive neurological disease with prominent psychiatric features. The clinical clue here is a vertical gaze palsy, but in contrast to case 1, particularly affects downgaze.

In summary, we presented 2 cases of prominent behavioral (autistic spectrum) and psychiatric (paranoid psychosis) presentations to highlight these possible manifestations of *ATP13A2*‐related disease. Further reports and studies will help clarify to what extent these psychiatric aspects expand the clinical spectrum of *ATP13A2* mutations and should find consideration in the workup of pediatric and adult patients from neurologist's and psychiatrist's perspectives.

## Author Roles

(1) Research Project: A. Conception, B. Organization, C. Execution; (2) Statistical Analysis: A. Design, B. Execution, C. Review and Critique; (3) Manuscript Preparation: A. Writing of the first draft, B. Review and Critique.

B.B.: 1A, 1B, 1C, 2B, 3A

J.D.: 1C, 2B, 3B

F.M.: 2B, 3B

R.G.: 2B, 2C, 3B

J.B.: 2B, 2C, 3B

K.P.B.: 1A, 3B

## Disclosures

### Ethical Compliance Statement

We hereby confirm that the present study conforms to the ethical standards and guidelines of the journal. The patients have given written and informed consent for online publication of their videos. IRB approval was not necessary for the present case series.

### Funding Sources and Conflicts of Interest

No specific funding was received for this work. The authors declare that there are no conflicts of interest relevant to this work.

### Financial Disclosures for the Previous 12 Months

B.B., J.D., R.G., and J.B. report no disclosures. F.M. is supported by the European Academy of Neurology Research Fellowship 2020. K.P.B. has received grant support from Welcome/MRC, NIHR, Parkinsons's UK, and EU Horizon 2020. He receives royalties from publication of the *Oxford Specialist Handbook Parkinson's Disease and Other Movement Disorders* (Oxford University Press, 2008), *Marsden's Book of Movement Disorders* (Oxford University Press, 2012), and *Case Studies in Movement Disorders–Common and Uncommon Presentations* (Cambridge University Press, 2017). He has received honoraria/personal compensation for participating as consultant/scientific board member from Ipsen, Allergan, and Merz and honoraria for speaking at meetings and from Allergan, Ipsen, Merz, Sun Pharma, Teva, UCB Pharmaceuticals, the American Academy of Neurology, and the International Parkinson's Disease and Movement Disorders Society.

## Supporting information


**Video S1.** The video shows case 1. Although cooperative and friendly, he has difficulties following even simple commands; throughout the examination, he has stereotypies such as body rocking, finger pointing, or other hand movements. He appears to be hypomimic and has a mild upgaze restriction. There is a suggestion of right more than left bradykinesia when he attempts finger tapping, and left more than right dysmetria on finger–nose testing. He has mild dystonic posturing of the hands, hyperreflexia, and a spastic gait.

**Video S2.** The video shows case 2. Her saccades show an upgaze restriction in the vertical plane, and she has frontalis overactivity. Finger–nose testing reveals left intention tremor and mild dysmetria on the left more than on the right. Finger tapping shows mild bradykinesia on the right more than left. There is dysdiadochokinesia (right more than left), and leg agility is hampered by cerebellar ataxia more prominent on the left, but slower movements on the right. She has hyperreflexia with brisk reflexes with pathological spread such as a crossed adductor response. The gait is characterized by a combination of cerebellar ataxia and spasticity.
